# Multivoxel codes for representing and integrating acoustic features in human cortex

**DOI:** 10.1016/j.neuroimage.2020.116661

**Published:** 2020-08-15

**Authors:** Ediz Sohoglu, Sukhbinder Kumar, Maria Chait, Timothy D. Griffiths

**Affiliations:** a School of Psychology, University of Sussex, Brighton, BN1 9QH, United Kingdom; b Institute of Neurobiology, Medical School, Newcastle University, Newcastle Upon Tyne, NE2 4HH, United Kingdom; c Wellcome Trust Centre for Human Neuroimaging, University College London, London, WC1N 3BG, United Kingdom; d Ear Institute, University College London, London, United Kingdom

**Keywords:** Auditory cortex, Parietal cortex, fMRI, Multivariate, Feature binding

## Abstract

Using fMRI and multivariate pattern analysis, we determined whether spectral and temporal acoustic features are represented by independent or integrated multivoxel codes in human cortex. Listeners heard band-pass noise varying in frequency (spectral) and amplitude-modulation (AM) rate (temporal) features. In the superior temporal plane, changes in multivoxel activity due to frequency were largely invariant with respect to AM rate (and vice versa), consistent with an independent representation. In contrast, in posterior parietal cortex, multivoxel representation was exclusively integrated and tuned to specific conjunctions of frequency and AM features (albeit weakly). Direct between-region comparisons show that whereas independent coding of frequency weakened with increasing levels of the hierarchy, such a progression for AM and integrated coding was less fine-grained and only evident in the higher hierarchical levels from non-core to parietal cortex (with AM coding weakening and integrated coding strengthening). Our findings support the notion that primary auditory cortex can represent spectral and temporal acoustic features in an independent fashion and suggest a role for parietal cortex in feature integration and the structuring of sensory input.

## Highlights

• This study used fMRI and multivariate analysis to examine acoustic feature coding
• In auditory cortex, frequency and AM rate were represented primarily independently
• In parietal cortex, multivoxel representation was exclusively integrated

## 1 Introduction

In structuring the auditory scene, the brain must carry out two fundamental computations. First, it must derive *independent* representations of component acoustic features so that task-relevant features can be prioritized and task-irrelevant ones ignored. Second, to solve the well-known “binding problem”, the brain must subsequently *integrate* these separated representations into a coherent whole so that the features of a relevant sound source can be tracked successfully in cluttered scenes. Whether representations of stimulus features are independent or integrated is a longstanding issue in psychology (Treisman and Gelade, 1980; Ashby and Townsend, 1986) and neuroscience (Di Lollo, 2012; Soto et al., 2018). Even when not explicitly framed using these terms, many questions concerning sensory systems can be formalized in terms of representational independence versus integration (Soto et al., 2018).

It is widely believed that auditory processing is hierarchically organized and that neural representations are progressively transformed from independent to integrated codes as sensory information ascends the auditory pathway (Rauschecker and Tian, 2000; Bizley and Cohen, 2013). Thus, while neurons in low-level regions might respond to single stimulus features, higher-level neurons should show more complex tuning properties and respond to conjunctions of features. Precisely where along this continuum human primary auditory cortex (and regions beyond) fit within this conception of the auditory system has been the subject of debate.

Based on presumed similarities with the visual system, early models proposed that representations in primary auditory cortex were primarily independent, instantiated as topographically organized “feature maps” (see Nelken et al., 2003). According to such accounts, the integration of features is a computation that should most reliably be observed in non-primary regions. However, animal physiology studies demonstrate highly nonlinear neural responses already at the level of primary auditory cortex, suggestive of an integrated coding scheme (deCharms et al., 1998; Nelken et al., 2003; Chi et al., 2005; Wang et al., 2005; Christianson et al., 2008; Atencio et al., 2009; Bizley et al., 2009; Sadagopan and Wang, 2009; Sloas et al., 2016). The extent to which this also applies in humans remains unclear. While there are many sources of human imaging evidence that are potentially relevant to this issue, particularly investigations of how low-level acoustic features and higher-level categories are represented in cortical activity (Davis and Johnsrude, 2003; Zatorre et al., 2004; Cusack, 2005; Kumar et al., 2007; Staeren et al., 2009; Leaver and Rauschecker, 2010; Teki et al., 2011; Giordano et al., 2013; Norman-Haignere et al., 2015; Overath et al., 2015; Allen et al., 2017), fewer studies have directly tested and quantified the extent of representational independence versus integration in human cortex.

**Fig. 1:**
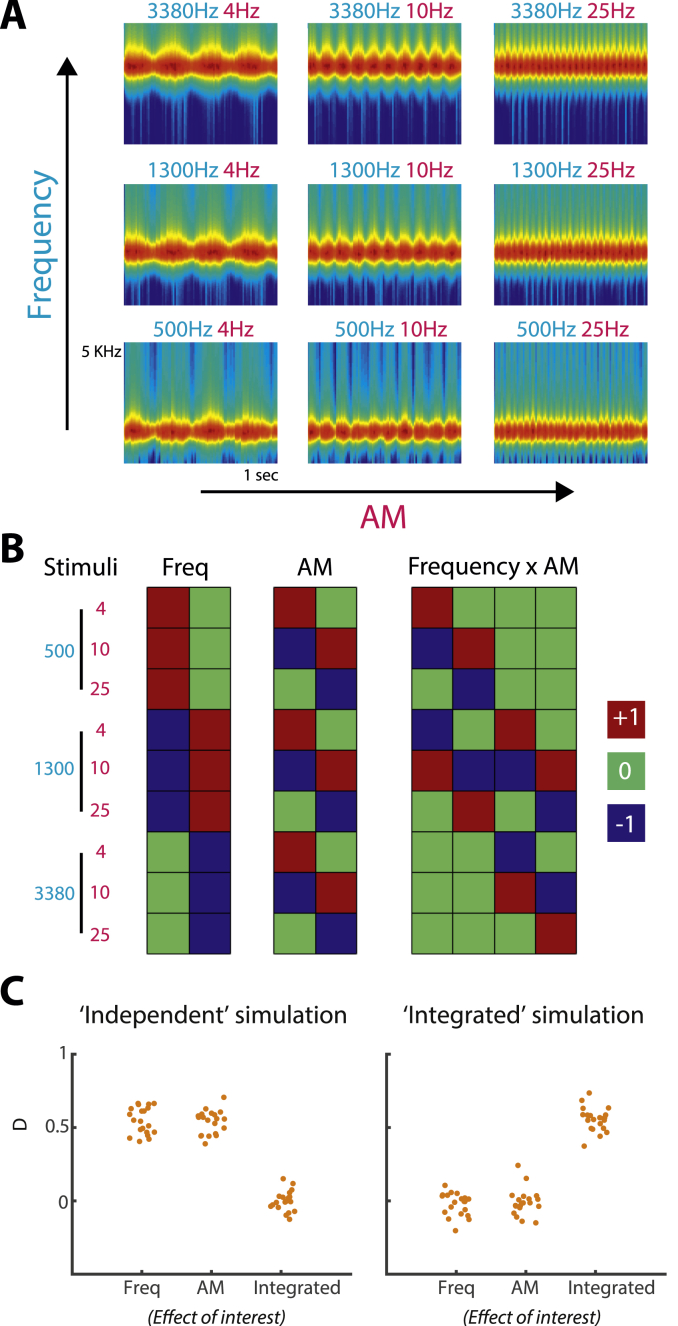
A) Spectrograms of the nine stimuli, with the spectrogram frequency axis equally spaced on a scale of Equivalent Rectangular Bandwidth (ERB; Moore and Glasberg, 1983) and smoothed to obtain a temporal resolution similar to the Equivalent Rectangular Duration (Plack and Moore, 1990). This depiction more accurately captures spectral representation in the ascending auditory system than a spectrogram with a linear frequency axis. Note that the carrier frequencies of the presented stimuli were equally spaced on a logarithmic (rather than ERB) scale. The cyan- and magenta-colored text above each spectrogram indicate the center carrier frequency and AM rate of the bandpass noise, respectively. B) Statistical contrast matrices for testing the two main effects (of Frequency and AM) and Frequency ​× ​AM interaction. These contrasts follow the standard form for the two main effects and interaction under a 3 ​× ​3 design (Henson and Penny, 2005). From these three contrasts, we could test for independent and integrated coding (see Methods section for details). C) Multivariate pattern distinctness estimates for each effect of interest, when activity patterns were simulated using an independent representation (left-side graph) or an integrated representation (right-side graph). Each data point represents the pattern distinctness for a single iteration (“participant”) of the simulation. Freq, Frequency. D, Pattern distinctness.

In the current study, we used fMRI and multivariate pattern analysis to determine the extent to which spectral and temporal acoustic features are represented by independent or integrated multivoxel codes and how those codes are expressed over the human cortical hierarchy. Participants listened to band-pass noise varying across stimuli in frequency (a spectrally-based feature) and amplitude modulation (AM) rate (temporally-based; see Fig. 1A). We chose to investigate these two acoustic features as they are sufficient alone to characterize much of the information present in biologically important sounds such as speech (Shannon et al., 1995; Roberts et al., 2011). An approach based on MANOVA (Allefeld and Haynes, 2014) allowed us to estimate the independent contributions of frequency and AM features to the observed multivoxel patterns, as opposed to nonlinear interactions between the features that are a signature of integrated coding (Kornysheva and Diedrichsen, 2014; Erez et al., 2015). Moreover, by acquiring whole-brain fMRI, we were able to characterize multivoxel representations across the entire human cortex, in contrast to more localized physiological recordings in animals.

## 2 Methods

### 2.1 Participants

Twenty participants (eleven female), aged between 18 and 27 years (mean ​= ​23, SD ​= ​2.4), were tested after being informed of the study’s procedure, which was approved by the research ethics committee of University College London. All reported normal hearing, normal or corrected-to-normal vision, and had no history of neurological disorders. Our sample size is in line with (or exceeds that of) related studies with *a priori* unknown effect sizes (e.g. Linke et al., 2011; Giordano et al., 2013; Allen et al., 2017; Santoro et al., 2017). While recent methods work recommends larger sample sizes for (univariate) fMRI studies (Geuter et al., 2018; Turner et al., 2018), we note that this recommendation was made in the context of more complex cognitive paradigms each lasting around 10 ​min. Thus, both cross- and within-participant variability might be expected to be greater than for the simpler sensory paradigm employed here conducted over a longer scanning time of 50 ​min (for a discussion of the trade-off between sample size and scan duration, see Nee, 2019).

### 2.2 Stimuli

The stimulus consisted of narrow (third of an octave) bandpass noise, amplitude modulated sinusoidally with 80% depth (see Fig. 1A). Each sound was presented for 1 ​s and varied across trials in center carrier frequency (from hereon, “frequency”) and amplitude modulation rate (“AM”). Frequency (500, 1300 and 3380 ​Hz) and AM (4, 10 and 25 ​Hz) were equally spaced on a logarithmic scale. Importantly for the purpose of assessing independent and integrated feature coding (see First-level statistics section below), frequency and AM varied across stimuli in an orthogonal fashion, such that every frequency was paired with every AM (i.e. nine stimuli in total, arranged as a 3 ​× ​3 factorial design). The relatively slow AM rates precluded the perception of pitch associated with the temporal modulation. In addition, the carrier center frequencies and bandwidths were chosen to avoid detectable spectral cues from resolved sidebands in the stimulus (Moore, 2003). Sidebands will be most detectable for sounds with fast AM rates and low carrier frequencies (Moore and Glasberg, 2001). In the current study, this corresponds to the stimulus with the 500 ​Hz carrier frequency and 25 ​Hz AM rate. However, the sidebands resulting from this stimulus (500 ​+ ​25 ​= ​525 ​Hz and 500–25 ​= ​475 ​Hz) fall inside the auditory filter centered at 500 ​Hz with an equivalent rectangular bandwidth (ERB) of 79 ​Hz (Moore and Glasberg, 1983).

Stimuli were matched in terms of their RMS amplitude and shaped with 20 ​ms raised-cosine onset and offset ramps. Bandpass noise was synthesized independently on each presentation (with a sampling rate of 44,100 ​Hz) and delivered diotically through MRI-compatible insert earphones (S14, Sensimetrics Corporation). To compensate for resonances in the frequency response of the earphones, the stimuli were digitally preprocessed using the filters and software provided with the earphones.

### 2.3 Procedure

Stimulus delivery was controlled with Cogent toolbox (http://www.vislab.ucl.ac.uk/cogent) in Matlab (MathWorks). Participants were scanned for five runs, each lasting around 10 ​min consisting of sixteen repetitions of the nine stimuli. For one participant, there was insufficient time to scan for the fifth run because of technical difficulties. Stimuli were grouped into blocks of eighteen sounds within which all nine stimuli appeared twice and in random order. The inter-stimulus interval ranged uniformly between 2000 and 4000 ​ms.

Participants were instructed to listen carefully to the sounds while looking at a central fixation cross and press a button (with their right hand) each time a brief (150 ​ms duration) white-noise interruption occurred during sound presentation. These white-noise interruptions were unmodulated in their amplitude profile and occurred on a small percentage (~6%) of stimuli (once every block of eighteen sounds). Group performance was near ceiling, confirming engagement with the task. The average hit rate was .98 (ranging from 0.8 to 1 across participants; SEM ​= ​0.014) with no false alarms.

To estimate the perceived saliency of the sounds, two participants from the main fMRI experiment and four new participants (two female; mean age ​= ​29 years, SD ​= ​4) completed a short behavioral session similar in procedure to Petsas et al. (2016). These participants listened to all pairwise combinations of the nine sounds (eight pairs for each of the nine sounds; separated by 200 ​ms of silence) and were asked to judge on each trial which of the two sounds was more salient. Participants were told that saliency refers to how much a sound would grab their attention. Pairs were presented three times in random order, with the order of the sounds within a pair counterbalanced across trials.

To estimate perceived loudness, we used the loudness model of Moore et al. (2016), as implemented in Matlab (http://hearing.psychol.cam.ac.uk/TVLBIN/tv2016Matlab.zip). As the model output differs slightly for different noise samples of the same stimulus, we generated an entire (single-participant) stimulus set in the same way as was done for the main experiment which we submitted to the model. We computed the time-varying long-term loudness, averaged over the duration of the stimulus and across noise samples for each of the nine stimuli.

### 2.4 Image acquisition

Imaging data were collected on a Siemens 3 ​T Quattro MRI scanner (http://www.siemens.com) at the Wellcome Trust Center for Human NeuroImaging, University College London. A total of 175 echo planar imaging (EPI) volumes were acquired per run, using a 32-channel head coil and continuous sequence (TR ​= ​3.36 ​s; TE ​= ​30 ​ms; 48 slices covering the whole brain; 3 ​mm isotropic resolution; matrix size ​= ​64 ​× ​74; echo spacing ​= ​0.5 ​ms; orientation ​= ​transverse). After the third run, field maps were acquired (short TE ​= ​10 ​ms; long TE ​= ​12.46 ​ms). During the functional scans, we also obtained physiological measures of each participant’s breathing and cardiac pulse. Because of technical issues, physiological measures were not available for two participants. The experimental session concluded with the acquisition of a high-resolution (1 ​× ​1 ​× ​1 ​mm) T1-weighted structural MRI scan.

The randomized presentation order of the nine stimuli was employed to sensitively detect between-stimulus differences in BOLD signal (Josephs and Henson, 1999). However, our experimental design also permitted detection of sound versus implicit baseline as we randomized the ISIs uniformly between 2 and 4 ​s (equivalent to 3–5 ​s stimulus onset asynchrony). Although this stimulus timing is fast relative to the duration of the haemodynamic response function (which peaks around 5 ​s), the randomization of ISIs sufficiently enabled the detection of BOLD signal variations relating to sound versus baseline. This is confirmed by inspection of the predicted BOLD timeseries and by the parameter estimates in superior temporal plane regions, which were reliably greater than baseline (shown in Figure 3).

**Fig. 2:**
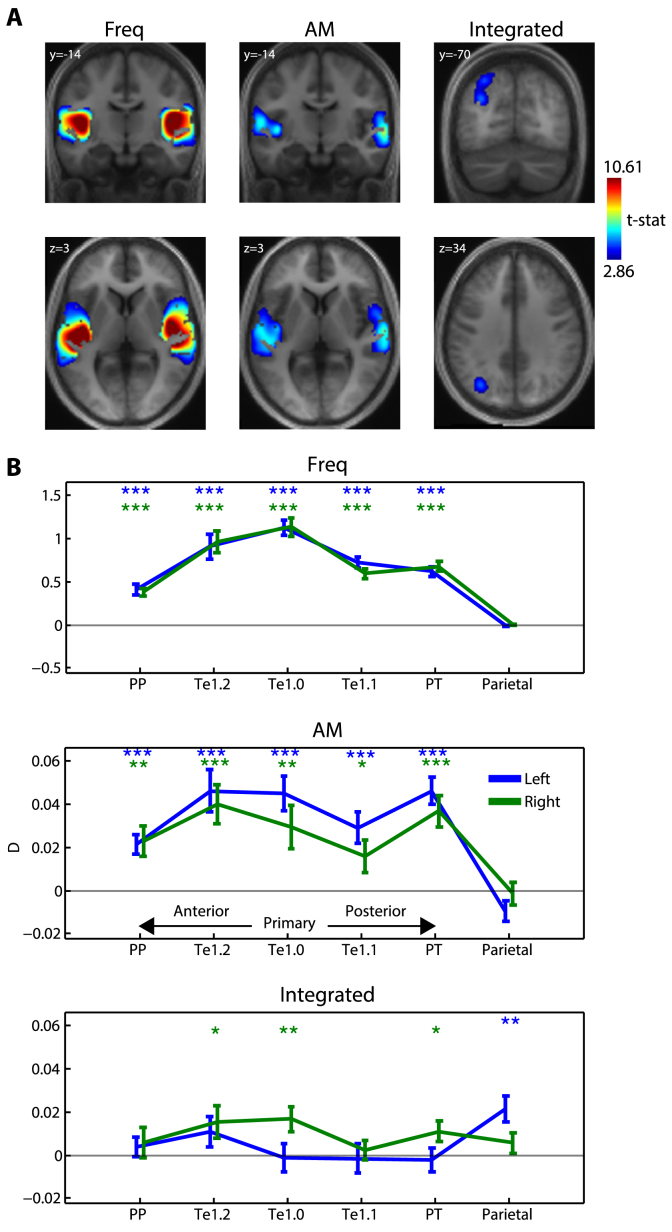
Whole-cortex multivariate searchlight analysis. A) Group-level statistical maps for each effect of interest, overlaid onto coronal and axial sections of the group-averaged structural (in MNI space) and thresholded voxelwise at *p* ​< ​.005 and clusterwise at *p* ​< ​.05 (FWE corrected for multiple comparisons). B) ROI analysis. Each data point shows the pattern distinctness D, averaged over the searchlight map within each ROI and over participants. Error bars represent the standard error of the mean. Asterisk symbols above each data point indicate significantly above-zero pattern distinctness, FDR corrected for multiple comparisons across contrasts, ROIs and hemispheres. ∗∗∗*p* ​< ​.001, ∗∗*p* ​< ​.01, ∗*p* ​< ​.05.

### 2.5 Image processing

fMRI analysis was performed in SPM12 (http://www.fil.ion.ucl.ac.uk/spm). After discarding the first three volumes to allow for magnetic saturation effects, the remaining images were realigned and unwarped to the first volume to correct for movement of participants during scanning. Also at the unwarping stage, the acquired field maps were used to correct for geometric distortions in the EPI due to magnetic field variations. Realigned images were co-registered to the mean functional image and then subjected to multivariate statistical analysis, generating searchlight maps from unsmoothed data in each participant’s native space (see First-level statistics section below). Searchlight maps were subsequently normalized to the Montreal Neurological Institute (MNI) template image using the parameters from the segmentation of the structural image (resampled resolution: 2 ​× ​2 ​× ​2 ​mm) and smoothed with a Gaussian kernel of 6 ​mm full-width at half-maximum. Where additional univariate analyses are reported, realigned images were spatially normalized and smoothed first before statistical analysis.

### 2.6 First-level statistics

Statistical analysis was based on the general linear model (GLM) of each participant’s fMRI time series, using a 1/128 ​s highpass filter and AR1 correction for auto-correlation. The design matrix comprised the auditory stimulus events, each modeled as a stick (delta) function and convolved with the canonical haemodynamic response function. Separate columns were specified for each of the nine stimuli, in addition to a column for target sounds (to remove variance associated with the white noise interruptions and the button presses). Additional columns were specified for the six movement parameters and the mean of each run. Cardiac and respiratory phase (including their aliased harmonics) as well as heart rate and respiratory volume were modeled using an in-house Matlab toolbox (Hutton et al., 2011). This resulted in fourteen physiological regressors in total: six each for cardiac and respiratory phase and one each for heart rate and respiratory volume.

For statistical inference, we used cross-validated multivariate analysis of variance (Allefeld and Haynes, 2014), as implemented in the cvMANOVA toolbox in Matlab (version 3; https://github.com/allefeld/cvmanova). For each participant, this method measures the pattern distinctness D, a cross-validated version of one of the standard multivariate statistics: Lawley-Hotelling’s trace.

Lawley-Hotelling’s trace (*Δ*_*LH*_) quantifies the amount of multivariate variance explainable by an experimental contrast, in units of error variance:

$$\Delta_{LH}=\frac{B_{\Delta }^{’}X^{’}XB_{\Delta }}{\sum }$$

where *B*_*Δ*_ are the parameter contrasts, *X* is the design matrix and *∑* is the error covariance matrix. The pattern distinctness D is derived by additionally cross-validating the data using a leave-one-run-out procedure (for further details, see Allefeld and Haynes, 2014). Cross-validation ensures that the expected value of D is zero if two voxel patterns are not statistically different from each other, making D a suitable summary statistic for group-level inference (e.g. with the one-sample *t*-test). Note that because of this cross-validation, D can sometimes be negative if its true value is close to zero in the presence of noise.

In contrast to classification accuracy from pattern decoders, which is dependent on the particular algorithm used as well as the amount of data and partitioning into training and test sets, D is a clearly interpretable, standardized effect size (for examples of previous applications, see Guggenmos et al., 2016; Christophel et al., 2017, 2018; Dijkstra et al., 2017). When applied to the simple case of only two stimuli, D is a measure of between-stimulus pattern dissimilarity and is closely related to the (cross-validated) Mahalanobis distance, which has been demonstrated to be a more reliable and accurate metric for characterizing multivoxel patterns than the correlation or Euclidean distance (Kriegeskorte et al., 2006; Ejaz et al., 2015; Walther et al., 2016). Like the Mahalanobis distance, D takes into account the spatial structure of the noise (GLM residuals) by normalizing the multivoxel variation for an experimental effect by the noise covariance between voxels. As D is obtained from the GLM, cvMANOVA can also be used to test more complex contrasts such as the main effects and interactions of a factorial design. For the 3 ​× ​3 design of the present study, the contrast matrices for the two main effects and interaction take the standard form (Henson and Penny, 2005) and are shown in Fig. 1B.

We tested the extent to which frequency and AM features are represented by independent or integrated multivoxel codes by examining three effects of interest. If frequency and AM features are represented in an integrated fashion, then changes in these two features should combine nonlinearly (non-additively) to influence multivoxel activity patterns (see Kornysheva and Diedrichsen, 2014; Erez et al., 2015). In other words, the effect of frequency should differ depending on AM (and vice versa). Thus, the first effect of interest was the interaction between frequency and AM and quantified the extent of integrated coding. If on the other hand, frequency and AM features are coded independently, then changes in these two features should result in a linear (additive) effect on activity patterns. An independent effect implies that changes in voxel patterns attributable to the frequency feature remain invariant with respect to AM (and vice versa): there is no interaction. Within the cvMANOVA framework, the extent of independence can therefore be quantified by subtracting the interaction from the main effects as follows (see equation 19 in Allefeld and Haynes, 2014):

$$Independent\;coding\;of\;frequency=D\left(Freq\right)-\frac{1}{L\left(Freq\right)-1}D\left(Interaction\right)$$

$$Independent\;coding\;of\;AM=D\left(AM\right)-\frac{1}{L\left(AM\right)-1}D\left(Interaction\right)$$

where *D(Freq)*, *D(AM)* and *D(Interaction)* are the pattern distinctness estimates for the main effects of frequency, AM and the interaction, respectively. *L(Freq)* and *L(AM)* are the number of levels for the frequency and AM factors, respectively (for the current design, *L(Freq)* ​= ​*L(AM)* ​= ​3). Allefeld and Haynes (2014) expressed such contrasts as measures of “pattern stability” but are equivalently considered measures of independent coding (Kornysheva and Diedrichsen, 2014).

Computational simulations confirm that the above effects of interest capture the presence of independent and integrated representations, in line with previous modeling work and applications in the visual and motor domains (Allefeld and Haynes, 2014; Kornysheva and Diedrichsen, 2014). For each of twenty “participants” and nine stimuli, we generated synthetic activity patterns over 123 voxels consisting of the true underlying pattern (normal random vector) and a noise component that was generated independently for each of five “runs” and sixteen repetitions of the nine stimuli. These synthetic data were then submitted to cvMANOVA resulting in a pattern distinctness estimate for each participant and effect of interest.

Two versions of the simulation were run, differing in the generative model used to produce the voxel patterns. In the first version, frequency and AM features were represented independently. That is, voxel patterns were generated separately for the two features and summed together to obtain voxel patterns (Y) for each of the nine stimuli with carrier center frequency f and AM rate m:

Y_f,m_ ​= ​F_f_ ​+ ​T_m_ ​+ ​e_f,m_

where *F* and *T* denote, respectively, the voxel pattern representations for the frequency and AM features and e the noise.

In the second version, frequency and AM were represented in an integrated fashion by generating a unique pattern for each of the nine stimuli. Thus, in this version of the simulation, the representation of frequency is inseparable from that of AM:

Y_f,m_ ​= ​FT_f,m_ ​+ ​e_f,m_

Here *FT* denotes the true pattern that was generated uniquely for each stimulus. In both versions, the resulting patterns were scaled to have the same mean and variance.

In the present version of the simulations, the variance of the noise was set to 10 times that of the true underlying pattern. We could vary this ratio to modulate the overall effect sizes in the simulations and to match those observed in the experimental data. However, our goal here was not to recreate the precise conditions of the experiment. This would require modeling the spatiotemporal correlation of within-subject noise and cross-subject variability, which is outside the scope of the current study. Rather, through the use of a generative model, we wished to provide a more formal definition of independent and integrated coding. In addition, we wished to confirm that *in principle*, our experimental contrasts can indeed capture independent and integrated coding in the specific context of our 3 ​× ​3 factorial design. For more detailed simulations that validate the current methods, see Allefeld and Haynes (2014) and Kornysheva and Diedrichsen (2014).

As Fig. 1C shows, when frequency and AM were simulated as independent representations, the pattern distinctness D was significantly greater than zero when testing the independent (but not integrated) coding effects of interest (frequency: *t*(19) ​= ​29.2, *p* ​< ​.001; AM: *t*(19) ​= ​35.1, *p* ​< ​.001; Integrated: *t*(19) ​= ​−0.104, *p* ​= ​.541). In contrast, when frequency and AM were represented in an integrated fashion, the reverse was true with a significant effect of integrated (but not independent) coding (frequency: *t*(19) ​= ​−1.39, *p* ​= ​.910; AM: *t*(19) ​= ​−0.429, p ​= ​.664; Integrated: *t*(19) ​= ​33.0, *p* ​< ​.001). This pattern of results was supported by a repeated measures ANOVA in which we observed a significant two-way interaction between simulation type (independent versus integrated) and effect of interest (frequency/AM/integrated; *F*(2,30) ​= ​737.2, *p* ​< ​.001).

cvMANOVA was performed as a searchlight analysis (Kriegeskorte et al., 2006) using spheres with a radius of three voxels (~9 ​mm; ~123 voxels of 3 ​× ​3 ​× ​3 ​mm) and constrained to voxels within the whole-brain mask generated by SPM during model estimation. This whole-brain mask does not explicitly exclude white matter voxels but inspection of the overlap with a probabilistic white matter mask revealed no overlap with high probability (>80%) white matter voxels. Moreover, the noise normalization performed by cvMANOVA should in principle automatically downweight noise from white matter voxels, circumventing the need to explicitly distinguish between gray and white matter. Thus, for each participant and effect of interest, a whole-brain searchlight image was generated in which each voxel expressed the pattern distinctness D over that voxel and the surrounding neighborhood. As recommended by Allefeld and Haynes (2014), to correct for searchlight spheres near the brain mask boundaries containing fewer voxels, the estimate of D at each voxel was adjusted by dividing by the square root of the number of voxels within the searchlight.

### 2.7 Group-level statistics

For whole-cortex statistical analysis of the multivariate data, searchlight images were submitted to a group-level one-sample *t*-test under minimal assumptions using the nonparametric permutation test (Nichols and Holmes, 2002). In this procedure, the sign of the pattern distinctness at each voxel for each subject was randomly flipped. The one-sample *t*-statistic was subsequently computed at each voxel, the image thresholded and the largest cluster size noted. By repeating these steps over a number of iterations (here 5000), we could build a null distribution of cluster sizes against which to compare the observed cluster size at each voxel. Note that because the true pattern distinctness can never be negative, a one-sample *t*-test in this context effectively provides fixed-effect inference (Allefeld et al., 2016). This is similar to *t*-tests on classification accuracies, the true values of which can never be below chance. Whole-cortex statistics for the univariate analysis were also based on the permutation test. Here we used a one-sample *t*-test for comparing sound-evoked activation with the implicit baseline and repeated measures ANOVA with the factors frequency and AM to test between-stimulus differences. When using ANOVA, the null distribution was created by randomly shuffling the nine stimulus labels. We constrained all analyses to voxels within the cortex (as defined by the probabilistic Harvard-Oxford cortical mask thresholded at 25%, distributed with FslView https://fsl.fmrib.ox.ac.uk). Statistical maps were thresholded voxelwise at *p* ​< ​.005 and clusterwise at *p* ​< ​.05 (familywise error [FWE] corrected for multiple comparisons).

Additional region of interest (ROI) analyses were conducted by averaging over the searchlight and univariate contrast images in locations anatomically defined by the Jülich and Harvard-Oxford probabilistic atlases (distributed with FslView) and thresholded at 30%. This ROI analysis was conducted parametrically (i.e. without using the permutation test). The ROIs included primary auditory cortex (area Te1.0 in middle Heschl’s gyrus [HG]) and the non-primary auditory areas Te1.1 (posteromedial HG), Te1.2 (anterolateral HG), planum polare (PP) and planum temporale (PT). We also tested the posterior parietal region revealed in the whole-cortex searchlight analysis, to enable a comparison of effect size with the auditory cortical ROIs and to statistically test for between-region differences. To avoid statistical “double-dipping” (Kriegeskorte et al., 2009), we used a leave-one-subject-out procedure (Esterman et al., 2010) in which the whole-cortex second level *t*-test was repeatedly re-estimated, each time leaving out one participant, and using the resulting left parietal cluster as the ROI for the left out subject (cluster defining threshold *p* ​< ​.005 uncorrected). To obtain the homologous cluster in the right hemisphere, each left parietal cluster was left-right flipped using MarsBaR toolbox for SPM (http://marsbar.sourceforge.net). This enabled us to statistically compare effects in parietal cortex with those in the superior temporal plane ROIs (which were distributed in both hemispheres). To reduce computation time, these leave-one-subject-out *t*-tests were also conducted parametrically in SPM. To facilitate interpretation, ROI effect sizes for the multivariate analysis are reported after transforming the adjusted pattern distinctness back into the original estimate (by multiplying by a constant factor of √123 i.e. the typical number of voxels within each searchlight).

Classical multidimensional scaling (MDS) was performed on single-participant dissimilarity matrices in selected ROIs. The resulting MDS solutions were averaged over participants after Procrustes alignment to account for the arbitrary rotation induced by the MDS procedure. Because Procrustes alignment potentially removes some of the true inter-individual differences, the standard error ellipses in Fig. 4B should be considered a lower-bound estimate of cross-participant variability (Ejaz et al., 2015). To further visualize the dissimilarity relationships, we subjected the dissimilarity matrices to an agglomerative hierarchical clustering procedure (based on complete-linkage) and visualized the results with dendrograms (see Nili et al., 2014). Dissimilarity matrices were formed by computing the pattern distinctness of all pairwise comparison contrasts between the nine stimuli and subjected to a group-level one-sample *t*-test. Given that the goal of this analysis was to better visualize effects of interest already identified as significant (i.e. the independent and integrated contrasts in the whole-cortex and ROI analyses), we thresholded these dissimilarity matrices at *p* ​< ​.05 uncorrected.

## 3 Results

### 3.1 Cortical distribution of independent and integrated codes

We used cross-validated MANOVA (Allefeld and Haynes, 2014) to determine the extent to which cortical activity patterns show evidence for 1) independent coding of frequency, in which the influence of frequency was invariant with respect to AM, 2) independent coding of AM, in which the influence of AM was invariant with frequency or 3) integrated coding, in which the influences of frequency and AM were interdependent. This was achieved by testing whether the pattern distinctness D over a searchlight sphere or ROI was significantly above zero for the independent and integrated effects of interest (see First-level statistics in the Methods section).

Using a whole-cortex searchlight analysis (Kriegeskorte et al., 2006), we detected large clusters in the superior temporal plane bilaterally (extending into the superior temporal gyrus) that showed significant independent coding of frequency and AM (Fig. 2A and Table 1). Within these regions of auditory cortex, there was no evidence for integrated coding after correcting for multiple comparisons over the whole cortex. Instead, significant integrated coding was observed in a cluster outside of classically defined auditory cortex in the left posterior parietal lobe, extending over the inferior and superior portions of the parietal lobule and the intraparietal sulcus.

**Table 1:** MNI coordinates and anatomical labels for significant multivariate searchlight effects.

Effect of Interest	Hemisphere	Region Label	Extent	t-value	x	y	z
Frequency	Left	Te1.0	6056	14.2923	-44	-22	6
		Superior Temporal Gyrus		13.1983	-48	-26	-2
		Superior Temporal Gyrus		6.2306	-54	-44	12
	Right	Rolandic Operculum	5987	14.0351	50	-20	14
		Superior Temporal Gyrus		12.1458	50	-22	0
		Te1.2		8.3851	58	-6	-4
AM	Left	Superior Temporal Gyrus	2721	7.9246	-50	-30	14
		Superior Temporal Gyrus		5.8524	-42	-14	0
		Superior Temporal Gyrus		5.1419	-52	-36	6
	Right	Superior Temporal Gyrus	2384	7.0587	62	-14	-2
		Superior Temporal Gyrus		5.7386	62	-28	8
		Inferior Frontal Gyrus		3.3739	56	12	10
Integrated	Left	Intraparietal Sulcus / Inferior Parietal Lobule	445	4.5942	-32	-68	32
		Superior Parietal Lobule		4.3273	-28	-72	50
Saliency	Left	Superior Temporal Gyrus	5446	17.963	-52	-30	8
		Superior Temporal Gyrus		8.139	-50	-16	-6
		Superior Temporal Gyrus		7.021	-58	-10	0
	Right	Superior Temporal Gyrus	5409	14.961	56	-24	8
		Te1.0		14.005	52	-16	4
		Insula		13.794	42	-14	0

We next conducted an ROI analysis in which independent and integrated coding was tested in anatomically defined regions in the superior temporal plane as well as the posterior parietal region identified in the whole-cortex searchlight analysis. We first tested each ROI separately, using false discovery rate (FDR) correction for multiple comparisons across 6 ROIs x 2 hemispheres x 3 effects of interest (Genovese et al., 2002). As expected from the earlier whole-cortex analysis, significant independent coding of both frequency and AM was observed in all auditory ROIs but not in posterior parietal cortex (shown in Fig. 2B). The effect size for independent coding of AM (mean D ​= ​0.02–0.04 over auditory regions) was smaller than for frequency, amounting to no more than 8% of the frequency effect size (mean D ​= ​0.5–1.0). Also expected was significant integrated coding in the left posterior parietal ROI. However, additional effects of integrated coding were observed in right primary auditory cortex (area Te1.0), right anterolateral auditory area Te1.2 and right PT. The effect size for integrated coding (mean D ​= ​0.01–0.02 over right Te1.0, Te1.2, PT and left parietal) was considerably smaller than that for independent coding (50% of the AM effect size and no more than 4% of the frequency effect size).

Thus, the ROI analysis above suggests that in the superior temporal plane, cortical activation patterns show a mixture of components: a strong independent code and a weak integrated code. In contrast in parietal cortex, only an integrated code is present. In support of this pattern of results, we conducted repeated measures ANOVA with representation type (frequency/AM/integrated), region (primary/nonprimary/parietal) and hemisphere as factors. We observed a significant interaction between representation type and region (*F*(4,76) ​= ​154, *p* ​< ​.001). No factors involving hemisphere were significant and so in subsequent comparisons, we averaged the data over hemispheres. To further characterize the representation type by region interaction, we separately assessed how the magnitude of independent and integrated coding changed along successive stages of the cortical hierarchy. For independent coding of frequency, there was a significant decrease in pattern distinctness in non-primary versus primary auditory cortex (*t*(19) ​= ​−12.2, *p* ​< ​.001). This was also the case for parietal versus non-primary auditory cortex (*t*(19) ​= ​−11.8, *p* ​< ​.001). The pattern was less clear-cut for independent coding of AM and integrated coding. Like the results for the frequency feature, there was a significant decrease in independent coding of AM in parietal versus non-primary auditory cortex (*t*(19) ​= ​−7.67, *p* ​< ​.001). However, the equivalent comparison for non-primary versus primary auditory cortex was not significant (*t*(19) ​= ​−1.21, *p* ​= ​.120). For integrated coding, there was an increase in parietal versus non-primary auditory cortex (*t*(19) ​= ​1.82, *p* ​< ​.05). However, there was no significant difference between non-primary and primary auditory regions (*t*(19) ​= ​−0.797, *p* ​= ​.218). In summary, although there was a clear and fine-grained change across hierarchical levels in the strength of frequency coding (primary vs. non-primary auditory cortex, non-primary auditory vs. parietal cortex), such a change for AM and integrated coding was less fine-grained and only evident in the higher hierarchical levels (non-primary vs. parietal cortex).

**Fig. 3:**
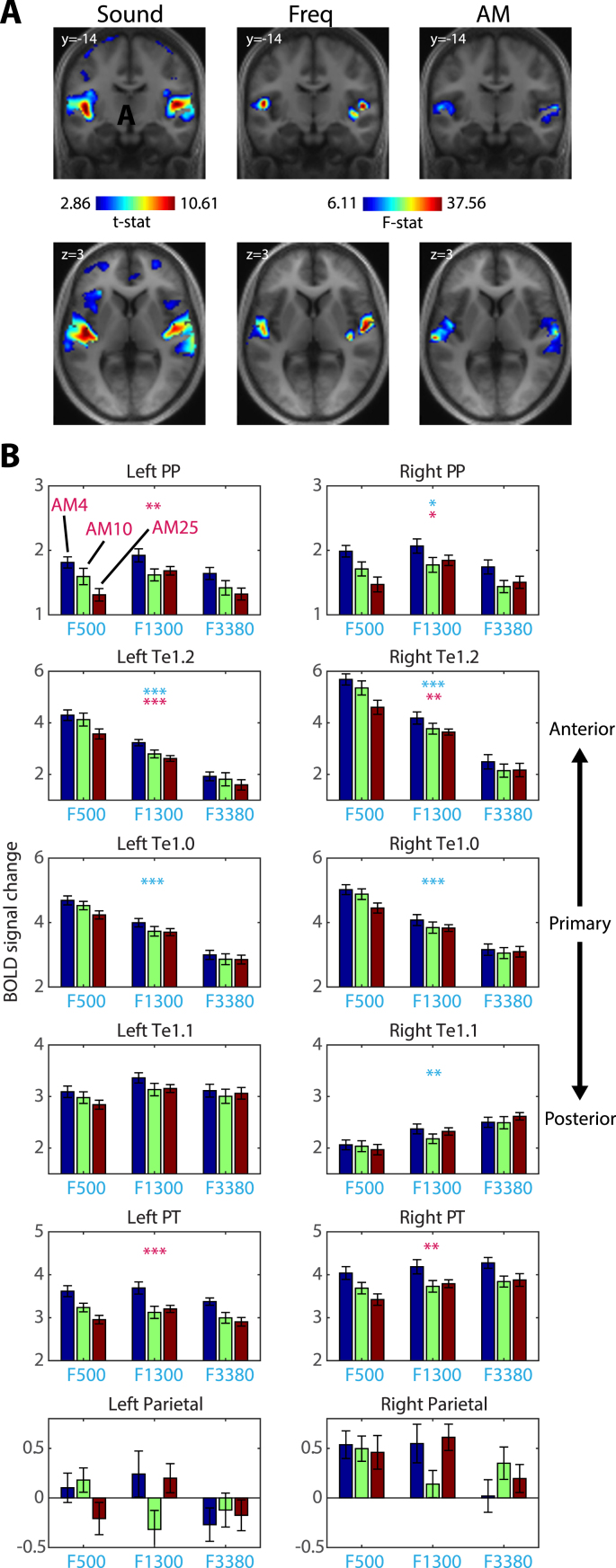
Univariate analysis. A) Whole-cortex analysis for contrasts of sound versus implicit baseline and main effects of frequency and AM. Images have been thresholded voxelwise at *p* ​< ​.005 and clusterwise at *p* ​< ​.05 (FWE corrected for multiple comparisons). B) ROI analysis. Data represent the BOLD signal change averaged over the spatial extent of each ROI and across participants. Error bars represent the standard error of the mean. Asterisk symbols indicate a significant main effect of frequency (in cyan) or AM rate (in magenta), FDR corrected for multiple comparisons across contrast, ROI and hemisphere. ∗∗∗*p* ​< ​.001, ∗∗*p* ​< ​.01, ∗*p* ​< ​.05.

Additional univariate analyses were conducted which were focused on the strength of activation. As expected, at the whole-cortex level, sound presentation was associated with increased BOLD responses in the superior temporal plane bilaterally (Fig. 3A and Table 2). No significant sound-evoked activations were observed in parietal cortex. Using repeated measures ANOVA (with frequency and AM as factors), we also evaluated between-stimulus differences in activation. Note that the main effects of frequency and AM for this analysis are conceptually different to the independent coding effects of the multivariate analysis. Here the main effect of frequency, for example, simply captures activation differences attributable to this factor rather than quantifying the extent of frequency invariance when AM rate changes. We observed significant effects of frequency and AM in the superior temporal plane bilaterally that survived whole-cortex testing but no significant frequency ​× ​AM interaction (Fig. 3A and Table 2). When conducting this analysis in the ROIs (shown in Fig. 3B), main effects of frequency and AM were present in auditory cortical regions but not in parietal cortex (FDR corrected as before, across 6 ROIs x 2 hemispheres x 3 effects of interest). Consistent with previous work (see Baumann et al., 2013; Moerel et al., 2014), follow-up *t*-tests in ROIs showing main effects showed a low carrier frequency preference in areas Te1.0 and Te1.2 bilaterally (all *p*’s ​< ​0.001) and a high carrier frequency preference in right Te1.1 (*t*(19) ​= ​3.46, *p* ​< ​.01). For the main effect of AM, the preference was for slow modulation rates throughout (all *p*’s ​< ​0.01; consistent with data from Overath et al., 2012). No significant interaction between frequency and AM was observed in any of the ROIs tested (even with an uncorrected threshold).

**Fig. 4:**
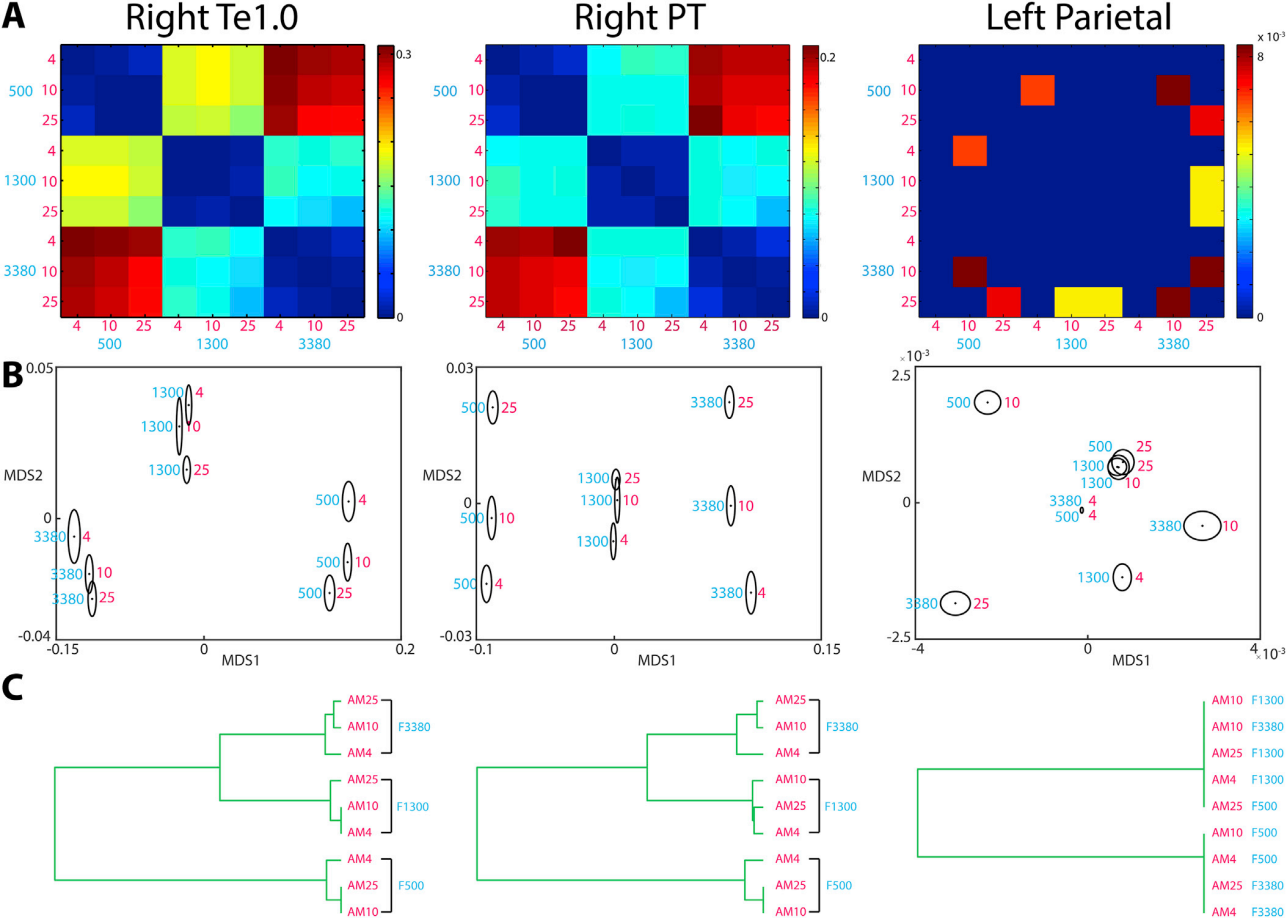
Visualizations of multivariate pattern distinctness A) Matrices expressing the multivoxel dissimilarity for all pairs of stimuli, averaged over the searchlight map within each ROI and over participants. Warm colors indicate multivoxel patterns that are highly dissimilar while cool colors indicate less dissimilarity. Dissimilarity matrices are shown thresholded based on a group-level one-sample *t*-test (see Walther et al., 2016) at *p* ​< ​.05 uncorrected. B) Group-averaged MDS solutions after Procrustes alignment across participants (first two dimensions plotted only). Each dot and surrounding ellipse represent the mean and its standard error, respectively. The cyan number beside each data point indicates the carrier center frequency of the bandpass noise while the magenta number indicates the AM rate. C) Dendrograms showing the results of hierarchical clustering.

**Table 2:** MNI coordinates and anatomical labels for significant univariate effects.

Effect of Interest	Hemisphere	Region Label	Extent	t-value	x	y	z
Sound	Left	Te1.0	4264	12.615	−50	−24	8
		Te1.0		11.616	−44	−18	6
		Te1.1		11.607	−42	−30	10
		Te1.0		10.689	50	−14	8
		Te1.0		9.493	48	−10	0
		Te1.1		9.389	44	−26	12
	Left	Medial Frontal	1841	5.251	−4	8	50
		Medial Frontal		4.904	0	14	46
		Anterior Cingulate		4.856	10	28	28
	Right	Middle Frontal Gyrus	703	4.317	26	52	6
		Middle Frontal Gyrus		4.065	34	46	14
		Middle Frontal Gyrus		3.988	38	34	34
	Left	Middle Frontal Gyrus	403	4.145	−32	40	22
		Middle Frontal Gyrus		4.118	−26	52	6
		Inferior Frontal Gyrus		3.436	−40	46	10
Frequency	Right	Te1.1	606	60.833	48	−28	10
		Te1.1		33.149	40	−20	2
		Superior Temporal Gyrus		31.023	46	−12	−6
	Left	Te1.0	666	50.659	−48	−20	8
		Te1.0		36.724	−52	−12	6
		Te1.2		20.665	−58	−6	2
	Right	Te1.0	443	40.95	52	−14	6
		Te1.2		39.995	54	−6	2
AM	Left	Superior Temporal Gyrus	1125	32.915	−62	−22	4
		Superior Temporal Gyrus		22.847	−66	−30	12
		Superior Temporal Gyrus		22.281	−52	−34	10
	Right	Superior Temporal Gyrus	877	25.747	62	−16	4
		Superior Temporal Gyrus		17.972	66	−28	8
		Te1.2		15.659	50	−6	−4

### 3.2 Multidimensional scaling and cluster analysis

Having established the cortical distribution of independent and integrated codes, we next used classical MDS to further characterize those codes (Kriegeskorte and Kievit, 2013). In three selected ROIs (right Te1.0, right PT and left parietal), we computed the pattern distinctness for all pairs of stimuli and assembled the results into dissimilarity matrices. These ROIs were chosen as together they fully sample the transition from auditory core to non-core to parietal cortex and show a mixture of independent and integrated coding profiles. As shown in Fig. 4A, we first averaged the matrices over participants and thresholded them based on a group-level one-sample *t*-test (see Walther et al., 2016). Given that we were interested in further characterizing independent and integrated effects previously shown as significant, we used an uncorrected *p* ​< ​.05 threshold. MDS was performed to project the multivoxel dissimilarity structure onto a simple two-dimensional space (Fig. 4B). In this visualization, stimuli that are close together are associated with similar multivoxel activation patterns while stimuli that are far from each other are associated with dissimilar patterns.

In right primary auditory cortex (area Te1.0) and right PT, frequency and AM features were automatically projected by the MDS solution onto separate dimensions, despite the method having no information as to the stimulus features. Frequency was carried by the first MDS dimension (shown as the x-axis in Fig. 4B) while AM was carried by the second dimension (y-axis). This is consistent with our previous observation of these regions representing frequency and AM in a largely independent manner.

In contrast to auditory cortex, MDS for the left parietal ROI did not clearly separate frequency and AM features. The MDS solutions instead suggest that activation patterns in this region were modulated by particular conjunctions of carrier frequency and AM rate (e.g. F500AM10 and F3380AM25). This is again consistent with our previous observation that parietal cortex is characterized solely by an integrated code.

Visual inspection of the MDS plots in superior temporal regions suggests that carrier frequency was the main driver of multivoxel pattern dissimilarity. That is, multivoxel patterns were most dissimilar when evoked by different carrier frequencies. Indeed, hierarchical clustering analysis showed that multivoxel dissimilarities clearly clustered according to carrier frequency in right Te1.0 and PT (Fig. 4C). In contrast in the left parietal ROI, this analysis failed to reveal a clear clustering. These results are consistent with the effect sizes for independent and integrated coding shown previously in Fig. 2B.

### 3.3 Saliency analysis

**Fig. 5:**
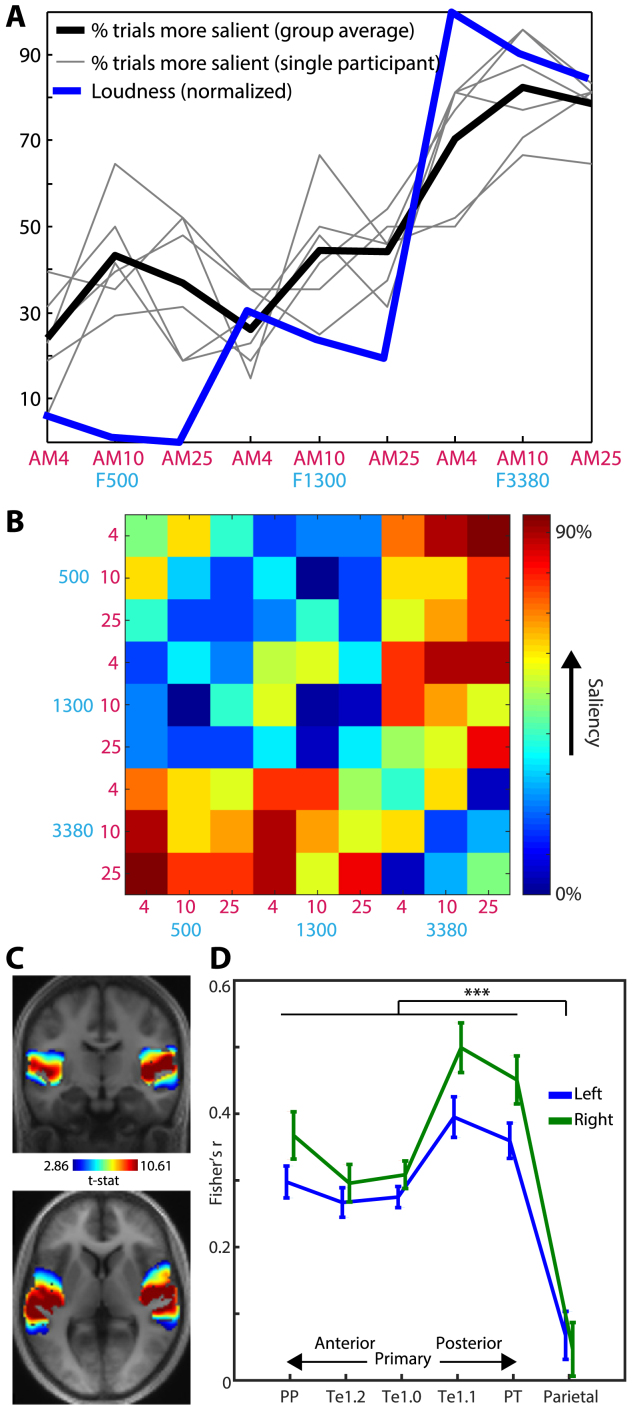
Saliency analysis. A) Subjective saliency of the stimuli. The thick black line indicates the group-averaged percentage of trials each stimulus was judged as more salient (than the other stimuli). Light gray lines indicate saliency judgements for individual participants. The thick blue line represents the predicted loudness of the stimuli according to the model of Moore et al. (2016) and normalized to have the same scale as the saliency data (for display purposes only). B) “Saliency distance” matrix expressing the absolute difference in the percentage of observations each sound in a pair was chosen as more salient. C) Whole-cortex multivariate searchlight analysis, showing where the Fisher transformed Spearman correlation between the saliency distance matrix in panel B and the multivoxel dissimilarity structure in each searchlight was significantly above zero across participants (thresholded voxelwise at *p* ​< ​.005 and clusterwise at *p* ​< ​.05 FWE corrected for multiple comparisons). D) ROI analysis. Each data point shows the Fisher transformed Spearman correlation, averaged over the searchlight map within each ROI and over participants. Error bars represent the standard error of the mean. Brace and asterisk indicates significant *p* ​< ​.001 ​*F*-test comparing the strength of Spearman correlation between auditory and parietal regions.

In the visual domain, parietal cortex has repeatedly been implicated in the processing of bottom-up saliency (Arcizet et al., 2011; Bogler et al., 2011). We therefore asked to what extent the integrated coding effect observed in posterior parietal cortex could be explained by between-stimulus differences in perceived saliency. In a separate behavioral session, listeners listened to all pairwise combinations of the nine sounds and judged which sound in each pair was more salient. We then estimated the perceived saliency of each sound as the percentage of trials the sound was chosen as more salient (shown in Fig. 5A as thick black line). Because saliency is related (although not identical) to loudness (Liao et al., 2015), we also show for comparison the loudness of the stimuli as predicted by the model of Moore et al., (2016) (shown in Fig. 5A as thick blue line).

Repeated measures ANOVA of the saliency judgments, with frequency and AM rate as factors, revealed a significant main effect of frequency (reflecting higher saliency for increasing frequency; *F*(2,10) ​= ​31.5, *p* ​< ​.001) and a significant main effect of AM rate (reflecting higher saliency for the middle AM rate; *F*(2,10) ​= ​6.34, *p* ​< ​.025). However, the interaction between frequency and AM rate was not significant (*F*(4,20) ​= ​0.808, p ​= ​.512). To directly test whether there was positive evidence for the null effect of no interaction, we also conducted repeated measures ANOVA as a Bayesian analysis (Rouder et al., 2016, 2017; Marsman and Wagenmakers, 2017). We contrasted a model which contained both main effects of frequency and AM and their interaction, with a null model that had the same structure but lacked the interaction (both models were assigned a prior probability of 0.5). This analysis indicated that the null model was 5 times more likely than the alternative model (Bayes Factor ​= ​5.31). As the integrated coding effect in parietal cortex is defined by the interaction between frequency and AM, the absence of an interaction in the saliency judgments is therefore inconsistent with a saliency-based account of the integrated coding effect in parietal cortex, or indeed, in any other of the regions in which integrated coding was observed.

As a further test of a saliency-based account, we used representational similarity analysis (RSA) to relate listeners’ saliency judgments to the observed multivoxel patterns (Kriegeskorte and Kievit, 2013). For each pair of sounds presented in the saliency judgment task, we pooled saliency judgments over trials and participants and computed the absolute difference in the percentage of observations each sound in the pair was chosen as more salient. From this we assembled a distance matrix quantifying the difference in saliency between the two sounds of all presented pairs (Fig. 5B). This “saliency distance” matrix provides a more detailed characterization of between-stimulus differences in saliency than the summary measure presented in Fig. 5A, which we could then correlate with the multivoxel dissimilarity matrix observed in each searchlight across the cortex of individual participants. As shown in Fig. 5C, the (Fisher-transformed) Spearman correlation between the saliency and multivoxel dissimilarity structure was significantly above zero in the superior temporal plane bilaterally but not in parietal cortex (for MNI coordinates, see Table 1). This pattern was further supported by an ROI analysis (Fig. 5D) in which the Spearman correlation significantly decreased from superior temporal to parietal cortex (*F*(1,19) ​= ​57.8, *p* ​< ​.001; effects involving hemisphere were not significant). We further note with interest how this saliency-to-multivoxel correlation peaked in posteromedial auditory area Te1.1, which clearly differs to how the independent and integrated coding effects were expressed over cortical regions (compare Fig. 5D with Fig. 2B). Nearly identical results were obtained when using loudness in this ROI analysis (here a loudness distance matrix was formed by computing the absolute differences in loudness between the stimuli). This suggests that saliency/loudness can be reliably dissociated from the independent and integrated coding effects of the earlier analyses. In summary then, this RSA analysis together with the absence of interactive influences of frequency and AM on behavioral saliency judgments suggests that the integrated coding effect we observe cannot be attributed to saliency/loudness. We will return to this point in the Discussion.

## 4 Discussion

In the current study, we manipulated two important acoustic features, frequency and AM rate, and determined the extent to which they are represented by independent versus integrated codes in fMRI multivoxel patterns. We demonstrate that these spectral and temporal dimensions are represented largely independently in the superior temporal plane, with only a weakly integrated component present in right Te1.0, Te1.2 and PT (amounting to no more than 4% of the frequency effect size and 50% of the AM rate effect size). In contrast, in a posterior parietal region not classically considered part of auditory cortex, multivoxel representation is exclusively integrated albeit weakly.

### 4.1 Independent representations in the superior temporal plane

Our demonstration of largely independent representations of frequency and AM rate in the superior temporal plane might seem to contrast with evidence from animal physiology that suggest highly nonlinear representations already at the level of primary auditory cortex (e.g. deCharms et al., 1998; Nelken et al., 2003; Wang et al., 2005). One explanation for why we see independent processing of frequency and AM is the spatial and temporal averaging inherent with fMRI (Heeger and Ress, 2002). This spatiotemporal averaging means that transient neural responses at a fine spatial scale will be underrepresented in BOLD signals and sustained responses at a large spatial scale will be overrepresented (Kriegeskorte and Diedrichsen, 2016; Guest and Love, 2017). Thus, while multivoxel patterns might show independent coding of frequency and AM, this does not exclude the possibility that other components of the neural representational code are nonlinear.

Our findings may also reflect the specific features that were manipulated. Specifically, it has been suggested that frequency and AM rate are fundamental dimensions of sound analysis (Dau et al., 1997; Chi et al., 2005) and in the auditory cortex are represented as orthogonally-organized topographic maps (“tonotopy” and “periodotopy”; e.g. Baumann et al., 2015). The presence of dissociable topographic maps might indicate that frequency and AM are two independent features to which the cortex is tuned. While previous electrophysiological (Langner et al., 2009) and fMRI (Baumann et al., 2015) findings from animals also support the notion of orthogonal topographic maps, in humans the evidence for an AM map is mixed with some studies showing clear topographic organization (Langner et al., 1997; Barton et al., 2012; Herdener et al., 2013) but others not (Giraud et al., 2000; Schönwiesner and Zatorre, 2009; Overath et al., 2012; Leaver and Rauschecker, 2016). Indeed, our univariate analysis showed regional preferences for slow modulation rates throughout (consistent with Overath et al., 2012) rather than a mixture of slow- and fast-tuned regions as would be expected for a topographic map (we acknowledge however that our analysis and imaging parameters were not optimized for characterizing modulation tuning using univariate methods; see below for further discussion). Previous conflicting findings may be attributed to the small size of auditory cortex and high inter-subject variability in anatomy. In the current study we circumvented these challenges by using a multivariate analysis method that abstracts away from the precise configuration of voxels. Importantly, this approach allowed us to directly test and quantify the degree of representational independence without the need to map features onto individual voxels.

Despite being able to robustly detect independent coding of frequency and AM rate in superior temporal regions, we nonetheless found a strong bias for the frequency feature, with the effect size for AM rate amounting to no more than 8% of the frequency effect size. While this result might indicate that superior temporal cortex is more strongly tuned to frequency, it could also reflect that AM rates in our study varied over a restricted range (4–25 ​Hz) in order to limit spectral confounds (Moore and Glasberg, 2001).

Independent representation of frequency and AM features is also suggested by component analysis of human fMRI responses to natural sounds (Norman-Haignere et al., 2015). This work suggests that frequency and AM features are represented as independent components in partly overlapping regions of the superior temporal plane. However, this study did not test for feature interactions between those features, leaving unclear the relative contributions of independent and integrated representations to neural responses. Another study that did test for feature interactions used forward encoding models to predict superior temporal fMRI responses to natural sounds from frequency and spectrotemporal modulations (Santoro et al., 2014). This work suggests that a model based on conjunctions of these features better predicted fMRI responses than if the features in the model were represented separately. While this result might be taken to be inconsistent with the highly independent code demonstrated here, we note that in our ROI analysis we too observed significant integrated coding in the superior temporal plane. But a consideration of the standardized effect sizes, which the MANOVA approach readily provides (Allefeld and Haynes, 2014), suggests a more nuanced interpretation. That is, while an integrated component may be necessary to fully explain fMRI responses (hence the superiority of an encoding model based on conjunctions of features), the majority of variance can be explained by an independent representation.

Thus, our study provides new evidence that frequency and AM are orthogonal dimensions of sound analysis. Such independent representation may support listeners’ ability to selectively process information in frequency versus time. In addition, as noted by Schnupp et al. (2001), an independent coding scheme will tend to convey more information than a highly-selective integrated code. This property would be desirable if the role of primary auditory cortex was to relay information to more specialized feature conjunction detectors in higher-level regions.

### 4.2 Integrated representation in posterior parietal cortex

Our imaging of the entire cortex allowed us to probe beyond classically defined auditory cortex. In this respect, a striking demonstration here is of an exclusively integrated representation of frequency and AM rate in a left posterior parietal region, at the border between the intraparietal sulcus (IPS), inferior parietal lobule and occipital cortex. This finding is notable for two reasons. First, it parallels findings from the visual domain in which parietal cortex (in particular the IPS) shows increased fMRI responses in feature conjunction versus single feature tasks (Donner et al., 2002; Shafritz et al., 2002; see also Baumgartner et al., 2013 for a similar finding using multivariate methods), with damage to this region leading to feature binding deficits (Humphreys et al., 2000). Second, BOLD activation in the IPS has been shown to systematically vary in auditory bi-stability (Cusack, 2005) and figure-ground paradigms (Teki et al., 2011, 2016). Indeed, the peak locations of the posterior parietal effects reported by these latter auditory studies fall inside the cluster reported here. In all these paradigms, perceptual outcomes are critically dependent on the way in which information across multiple features is combined and structured into object-based representations. Thus, the integrated representation for frequency and AM we observe here in parietal cortex is consistent with previous work suggesting a role for the IPS in feature integration and the structuring of sensory input. Further consistent with this, the location of our parietal cluster resides in the posterior portion of parietal cortex, where feature integration can be dissociated from effects of attention switching and task difficulty in anterior parietal regions (Cusack et al., 2010). However, our study goes beyond previous work in that neural responses evoked by stimulus features were contrasted directly, independently of listeners’ task (cf. feature search and bi-stability paradigms) and in the absence of salient stimulus features that would likely attract attention (cf. figure-ground paradigms).

Because of previous findings from the visual domain implicating parietal cortex in bottom-up saliency (Arcizet et al., 2011; Bogler et al., 2011), we also asked a separate group of listeners to rate the subjective saliency of the stimuli. While the sounds clearly differed in their subjective saliency, we found that influences of frequency and AM on the saliency ratings combined independently without evidence for an interaction, an observation inconsistent with a saliency-based account. It should be noted however that a limitation of this analysis is the small sample of participants (N ​= ​6) who provided the saliency judgments. In this respect, it is reassuring that when using RSA to relate saliency judgments to the dissimilarity structure of the multivoxel patterns, we found that the effect of saliency was confined to superior temporal plane regions with a peak in posteromedial auditory area Te1.1, which is reminiscent of findings by Behler and Uppenkamp (2016) who reported correlates of loudness in this region (see Liao et al., 2015 for the close relationship between loudness and saliency). Thus, the results from this saliency analysis suggest that the observed integrated coding effect does not appear to relate to bottom-up saliency.

Related to the issue of saliency, we also consider the possibility that the integrated coding profile we observe in parietal cortex was in part a consequence of listeners’ task. In our study, listeners performed an attentionally undemanding task that did not require explicit integration of frequency and AM features: detecting the target white-noise interruptions could in principle be based on changes in either the amplitude or spectral profiles alone. Despite this, one might argue that participants nevertheless detected the noise interruptions by attending to changes in both temporal and spectral content, in turn contributing to the integrated coding effect we observe. Indeed, as discussed below, attention has long been proposed to mediate feature integration (Treisman and Gelade, 1980). However, we think that this is unlikely as an explanation for the current findings. The interaction between frequency and AM rate in parietal cortex resulted from differences in the multivoxel patterns evoked by our stimuli (while the task was fixed throughout). Thus, even if listeners monitored both spectral and temporal content to detect the target interruptions, it is unclear how this would have preferentially biased listeners’ attention towards certain feature conjunctions. This is because the targets were temporally unmodulated and spectrally wide-band and therefore “neutral” with respect to the nine feature conjunctions of the stimuli.

A key assumption in our approach to distinguishing independent and integrated representations is a linear relationship between underlying neural activity and the measured fMRI signal (Kornysheva and Diedrichsen, 2014; Erez et al., 2015). Our univariate analysis shows that the mean signal amplitude in the posterior parietal region did not differ from the implicit baseline (or interstimulus period). It also did not differ between stimuli, neither in terms of mains effects nor in the interaction between frequency and AM rate. This suggests that our experimental manipulations in this region did not evoke sufficiently large changes in mean signal to saturate the fMRI response and produce nonlinear signal changes that could be misinterpreted as an integrated representation. Nonetheless, it should be noted that our rapid event-related design means that any parietal responses would not have had time to fully return to baseline between sound events. Thus, we cannot completely rule out the possibility that parietal regions were constantly active and operating near saturation. However, further evidence against saturation-driven nonlinearities comes from a recent study formally demonstrating that between-stimulus (and between-action) differences in multivoxel patterns are robust to large changes in mean activity levels (Arbuckle et al., 2019).

The integration of multiple feature representations is critical for building a cohesive perception of the auditory scene. However, even in parietal cortex, the effect size for integrated coding was small in comparison with that observed for independent coding in the superior temporal plane. Why then do we observe only weak integration of frequency and AM rate? As discussed above, frequency and AM may be privileged dimensions of sound analysis that are separable in a way that other dimensions are not. Our results may also be attributed to listeners performing an attentionally undemanding task that did not require explicit integration of frequency and AM features. It has been suggested that while individual features are detected automatically, feature integration is a computationally demanding process requiring focused attention (Treisman and Gelade, 1980; Shamma et al., 2011). Thus, the absence of focused attention to feature conjunctions could explain the weak integration we observe. Future work, using manipulations of attention, will be required to test this proposal.

### 4.3 Spatial resolution of current fMRI data and relationship with previous mapping studies

Because we wished to measure whole-brain responses, including in regions outside classically defined auditory cortex, we measured BOLD responses with a resolution of 3 ​mm isotropic voxels (the data were additionally smoothed with a 6 ​mm kernel but only after the critical multivariate statistics were computed). While finer-resolution data are commonly obtained in studies investigating how frequency and other acoustic features are mapped to individual voxels (e.g. Formisano et al., 2003; Barton et al., 2012; Herdener et al., 2013; Leaver and Rauschecker, 2016), our focus here is how frequency and AM features are represented in activity patterns over multiple voxels. It is well-established that multivoxel methods can sensitively measure changes in brain responses to acoustic features (even with 3 ​mm resolution data) by pooling weak but consistent signals over voxels and exploiting between-voxel correlations (e.g. Linke et al., 2011).

Note also that while significant independent coding of frequency and AM might be consistent with separate underlying neural populations responding to those features, this need not be the case. That is, the same neurons could simply be responding in a linear (additive) fashion to changes in frequency and AM rate. Moreover, the extent of representational independence versus integration does not bear on the issue of whether the underlying neural populations are “distributed” or “sparse” in nature (Bizley and Cohen, 2013; Diedrichsen and Kriegeskorte, 2017). Thus, the extent of representational independence and integration in multivoxel patterns is a more abstract characterization of cortical processing than the precise spatial configuration of feature-tuned voxels.

### 4.4 Generality of findings

One question that arises from the current work is the extent to which our findings generalize to other acoustic features. Our factorial design, combined with synthetic stimuli, allowed us to orthogonalize changes in frequency and AM features in a controlled fashion. This is a statistically powerful method for dissociating contributions of experimental manipulations (here of acoustic features) to observed neural responses (Friston et al., 1994). At the same time however, this necessarily constrained the number of features we could investigate. Therefore, our findings should not be taken to mean that all acoustic features are encoded in the same way as the frequency and AM features studied here.

Our factorial design contrasts with studies that have investigated the neural representation of acoustic features using natural sounds and statistical methods that enable many stimulus features to be studied simultaneously (Giordano et al., 2013; Santoro et al., 2014; Di Liberto et al., 2015; Norman-Haignere et al., 2015; de Heer et al., 2017; Holdgraf et al., 2017; Brodbeck et al., 2018; Daube et al., 2019; Sohoglu, 2019). However, the benefits of this more naturalistic approach come with substantial methodological challenges since acoustic features in natural sounds show substantial correlations, making it difficult to dissociate their neural contributions (Holdgraf et al., 2017; Norman-Haignere and McDermott, 2018). Thus, we suggest that the approach taken here is complementary to studies using natural sounds. An extension for future work could increase the number of features manipulated factorially, combined with stimulus synthesis techniques to create more naturalistic (yet still controlled) sounds (Kawahara et al., 1999; McDermott and Simoncelli, 2011).

## CRediT authorship contribution statement

**Ediz Sohoglu:** Conceptualization, Methodology, Investigation, Data curation, Formal analysis, Writing - original draft, Writing - review & editing. **Sukhbinder Kumar:** Conceptualization, Methodology, Investigation, Writing - review & editing. **Maria Chait:** Conceptualization, Writing - review & editing. **Timothy D. Griffiths:** Conceptualization, Methodology, Funding acquisition, Writing - review & editing.
